# Silent Pauses and Speech Indices as Biomarkers for Primary Progressive Aphasia

**DOI:** 10.3390/medicina58101352

**Published:** 2022-09-27

**Authors:** Constantin Potagas, Zoi Nikitopoulou, Georgia Angelopoulou, Dimitrios Kasselimis, Nikolaos Laskaris, Evie Kourtidou, Vasilios C. Constantinides, Anastasia Bougea, George P. Paraskevas, Georgios Papageorgiou, Dimitrios Tsolakopoulos, Sokratis G. Papageorgiou, Elisabeth Kapaki

**Affiliations:** 1 Neuropsychology and Language Disorders Unit, 1st Department of Neurology, Eginitio Hospital, National and Kapodistrian University of Athens, 115 28 Athens, Greece; 2 Department of Speech and Language Therapy, School of Health Sciences, University of Peloponnese, 241 00 Kalamata, Greece; 3 Department of Psychology, Panteion University of Social and Political Sciences, 176 71 Athens, Greece; 4 Department of Industrial Design and Production Engineering, School of Engineering, University of West Attica, 122 43 Athens, Greece; 5 1st Department of Neurology, Eginitio Hospital, National and Kapodistrian University of Athens, 115 28 Athens, Greece; 6 2nd Department of Neurology, School of Medicine, National and Kapodistrian University of Athens, Attikon University Hospital, 115 28 Athens, Greece

**Keywords:** primary progressive aphasia, connected speech, silent pauses, speech rate, articulation rate

## Abstract

*Background and Objectives*: Recent studies highlight the importance of investigating biomarkers for diagnosing and classifying patients with primary progressive aphasia (PPA). Even though there is ongoing research on pathophysiological indices in this field, the use of behavioral variables, and especially speech-derived factors, has drawn little attention in the relevant literature. The present study aims to investigate the possible utility of speech-derived indices, particularly silent pauses, as biomarkers for primary progressive aphasia (PPA). *Materials and Methods*: We recruited 22 PPA patients and 17 healthy controls, from whom we obtained speech samples based on two elicitation tasks, i.e., cookie theft picture description (CTP) and the patients’ personal narration of the disease onset and course. *Results*: Four main indices were derived from these speech samples: speech rate, articulation rate, pause frequency, and pause duration. In order to investigate whether these indices could be used to discriminate between the four groups of participants (healthy individuals and the three patient subgroups corresponding to the three variants of PPA), we conducted three sets of analyses: a series of ANOVAs, two principal component analyses (PCAs), and two hierarchical cluster analyses (HCAs). The ANOVAs revealed significant differences between the four subgroups for all four variables, with the CTP results being more robust. The subsequent PCAs and HCAs were in accordance with the initial statistical comparisons, revealing that the speech-derived indices for CTP provided a clearer classification and were especially useful for distinguishing the non-fluent variant from healthy participants as well as from the two other PPA taxonomic categories. *Conclusions*: In sum, we argue that speech-derived indices, and especially silent pauses, could be used as complementary biomarkers to efficiently discriminate between PPA and healthy speakers, as well as between the three variants of the disease.

## 1. Introduction

Primary progressive aphasia (PPA) is a neurological syndrome that occurs as a result of frontotemporal lobar degeneration [1] or Alzheimer’s disease pathology [2,3]. Its main characteristic is a progressive decline in language functions, whereas the other cognitive domains remain relatively unaffected, at least during the first stages of the disease [1,3]. Thus, it is differentiated from the phenotype of typical Alzheimer’s disease (AD), which is related with prominent memory deficits and behavioral variant frontotemporal dementia which is mostly accompanied by behavioral disturbances [1].

The classical taxonomy introduced by the seminal paper of Gorno-Tempini and colleagues [3] suggests three PPA variants, each one related to distinct clinical characteristics as well as different areas of brain atrophy and discrepancies in the underlying pathology [4]. Non-fluent/agrammatic-variant PPA (nfvPPA) usually appears with effortful, agrammatic speech output and the impaired comprehension of complex syntactical structures, mostly due to predominant atrophy in the premotor and inferior frontal, anterior insula, and anterior part of the superior temporal regions of the left hemisphere and tau-positive pathology [3,5,6]. The logopenic variant is also considered a non-fluent variant, but it has a different language profile: patients usually exhibit word-retrieval deficits in both spontaneous speech and confrontation naming, as well as difficulties in sentence repetition. These individuals generally present atrophy in the left temporo-parietal regions of the brain and a high prevalence of amyloid-β and tau in the CSF compatible with AD pathology, while specific patterns of hypometabolism on F-18-fluorodeoxyglucose (FDG) PET have been reported [3,7,8,9,10]. Finally, the semantic variant, initially presenting as semantic dementia [11,12], is predominantly characterized by impaired single-word comprehension and deficits in confrontation naming due to limited access to semantic representations, whereas repetition and motor speech abilities are preserved [13,14]. Patients usually present increased cortical atrophy in temporal areas, such as the left anterior temporal lobe and temporal pole [15,16,17], often linked with TDP43-positive pathology [3].

Despite the aforementioned categorization, which is broadly used in clinical practice and research, there are a considerable number of patients who remain unclassified. Patients either present a single language difficulty for a long time or an atypical combination of language deficit features [3]. This cohort is usually referred to as mixed-PPA [18]. Gorno-Tempini and colleagues [3] suggest that classification criteria could be better implemented at the early stages of the disease. However, this is not always applicable, as patients do not always visit a clinician early enough for the initial manifestations of the disease to be directly assessed. More recent evidence highlights the problem of unclassified patients (see for example: [4,19,20,21,22,23,24]), indicating that these patients may present left-lateralized yet less extensive atrophy in language-related areas [19,20], as well as language deficits in more than one domain [20]. Moreover, Mesulam and colleagues [4] proposed that the same pathology may induce more than one variant and that the same PPA variant can be caused by more than one neuropathological entity, while in other studies it has been stated that dual pathologies may exist within the same patient [21]. It should be noted that additional categories, such as primary progressive anomic aphasia, primary progressive transcortical sensory aphasia, and primary progressive Wernicke’s aphasia, have also been proposed [22], suggesting that the PPA taxonomy should be reconsidered [19,20,23,24]. The taxonomy issue is highly related to the time of diagnosis. According to Mesulam and colleagues [4], the PPA phenotype may change over time, leading to considerable shifts in subtype classification; thus, PPA classification is dependent on the time post-onset of initial symptomatology. Based on a recent review of longitudinal studies on PPA, among the three PPA variants, logopenic patients tend to exhibit a gradual yet more intense deterioration of other cognitive domains, such as episodic memory, while non-fluent patients present a more robust profile of deficits in language abilities [25]. The distinct longitudinal changes in language and further neuropsychological deficits in PPA variants render the issue of diagnosis and taxonomy even more problematic.

The implementation of a wide range of standardized language assessment tools (for instance: confrontation naming tests, sentence completion, and sentence repetition tasks) has significantly contributed to the delineation of speech and language deficits and the categorization of PPA patients. However, connected speech evaluation via narrative tasks has also been proven valuable for the assessment of speech output capacity and inner language sub-processes, such as access to semantic and phonological representations, word retrieval, and sequencing via syntactical structure (see for example: [26,27,28,29,30]). Moreover, additional aspects such as the frequency of paraphasias (speech errors) have been shown to provide useful diagnostic information for the three PPA variants [31]. The temporal aspects of speech (more specifically, the frequency and duration of silent pauses) have been considered as indices of significant importance, revealing the internal cognitive processes that occur while speaking, such as access to semantic representations, the retrieval of the correct target word, monitoring, the selective retrieval of information from episodic/semantic memory, and the planning and organization of speech output [32,33,34,35,36]. There is increasing evidence indicating disturbances in specific patterns of silent pauses in clinical populations with acquired language deficits, such as post-stroke aphasia [35,37,38], patients with early Alzheimer’s disease [36,39], psychiatric disorders [40], and PPA. The latter studies aim to differentiate PPA variants [30], reveal selective deficits in word retrieval [41], capture distinct patterns of longitudinal changes in speech output [42], and highlight differences between patients with PPA and patients with Alzheimer’s disease [43]. In this sense, silent pauses may be considered as a biomarker that allows the distinction between healthy speakers and patients with acquired language disturbances, based on studies in both post-stroke aphasia patients [35] and patients with PPA [30], while they could also facilitate the differentiation of expressive language profiles among different patient profiles [30,38]. Moreover, it has been hypothesized that silent pauses may reflect a compensatory mechanism for word-finding deficits in narrative flow, and not only in patients with PPA [41]. A similar hypothesis has also been discussed for patients with Alzheimer’s disease [39], as it has been found that the frequency of silent pauses in narrative tasks has a significant positive relationship with neuropsychological tasks, reflecting word-finding ability. In addition to its suggested clinical utility, this approach, based on connected speech quantification, may be considered as a more ecological method than the standardized neuropsychological tasks that are used to detect possible language deterioration [35,39].

Finally, there is a general, yet not extensively studied, notion that distinct narrative tasks rely on certain linguistic demands, representing separate cognitive tasks of various levels of difficulty [44,45,46,47]. Therefore, several indices derived from connected speech quantification may be differentiated on the basis of the elicitation technique implemented [48]. Although there is some scarce evidence regarding PPA patients’ performance in elicitation tasks involving distinct speech genres [49,50,51], to our knowledge, no previous study has directly investigated the frequency and duration of silent pauses in diverse narrative tasks as possible biomarkers to differentiate PPA profiles.

The scope of the present study is twofold. First, we aimed to investigate the possible differences between patients with PPA and healthy controls according to indices derived from connected speech in two distinct narrative tasks, i.e., a picture description and a personal narration in which patients were asked to describe the course of their deficits. These indices were the frequency and duration of silent pauses, along with two other speech output metrics, the speech rate and articulation rate. Second, we aimed to investigate whether any of the above indices could serve as factors for categorizing patients into the three PPA variants. In sum, the main hypothesis of the present study was that pause metrics and speech-derived indices could serve as biomarkers for the diagnosis of PPA. Within this context, we aimed to use these markers to differentiate healthy individuals from PPA patients and to distinguish between the three PPA variants, i.e., logopenic, nonfluent, and semantic.

## 2. Materials and Methods

This was a case-control study conducted at the FIrst Department of Neurology of Eginition Hospital, National and Kapodistrian University of Athens, Greece.

### 2.1. Participants

Twenty-two patients (14 males) with PPA, who were 52–84 years old (mean age: 66.13) and had 2–18 years of formal schooling (mean years: 13.4), and 17 healthy speakers (7 men), who were 53–65 years old (mean age: 58.76) with 6–20 years of formal schooling (mean years: 14.58) (see Table 1 for participants’ demographics), were recruited from the First Neurological Clinic, Eginition Hospital. Patients’ neurological assessments and neuropsychological evaluations were conducted according to a previous study from our clinic [52]. Healthy speakers were recruited from the project “Investigation of cortical surface patterns and their relation with speech metrics and performance in neuropsychological assessment in healthy participants”, conducted at Eginition Hospital, School of Medicine, Greece (research protocol approval ID: ΩOΞΛ46Ψ8N2−7PN, July 2017). Data from this project have been used in other studies published by our group; however, this was the first time we used the specific variables as a reference point in contrast to PPA patients’ performance. Informed consent was obtained from all participants prior to participation, according to the Eginition Hospital Ethics Committee.

### 2.2. Language Assessment

Patients’ language ability was examined by the clinicians of the Neuropsychology and Language Disorders Unit at the First Department of Neurology of Eginition Hospital (EK, GA, DT, GP, and DK), who have extensive experience in the assessment of patients with aphasia, using the Boston Diagnostic Aphasia Examination standard assessment (BDAE-SF) [53], translated into Greek [54], along with other language tests, such as confrontation naming, receptive vocabulary, the comprehension of complex commands, verbal fluency, and word and pseudoword reading fluency measures, as presented in a previous work [55]. For the purposes of the study, the two standard elicitation tasks (patient story narration and “cookie theft” picture description) from BDAE-SF were used to obtain speech samples from each participant. Such elicitation tasks have already been used in research on PPA patients—see, for example [27,29,30]. For the picture description, each participant was asked to describe the cookie theft picture. For the patient story narration, patients were specifically requested to describe the course of their disease, whereas healthy participants were asked to describe a health issue/condition/ailment experienced by themselves or someone close to them, similar to the patient story [35,56]. All patients were classified into the three PPA taxonomic categories according to the clinical assessment, language profile, and overall cognitive performance, following the criteria of Gorno-Tempini and colleagues [3], by a group of experienced neurologists, neuropsychologists, and one speech-language therapist. Eight patients were classified as logopenic, six as semantic, and six as non-fluent. Two patients remained unclassified. See Table 2 for patients’ demographics, clinical characteristics, and performance in the standard neuropsychological assessment.

### 2.3. Speech Analysis

Speech samples were recorded and transcribed based on guidelines for discourse transcription in Greek [57]. The first 100 words uttered were isolated, and the silent pauses and speech duration were annotated, following a specific pipeline, previously implemented in speech samples of patients with post-stroke aphasia [35]. Filled pauses were annotated only to be removed from any further analysis, as they were unrelated to the scope of the current study. The frequency of pauses (pauses per 100 words) and the total duration of pauses adjusted for the total number of words were calculated. Moreover, the speech rate was calculated by dividing the total number of words by the total duration of the speech sample (including all disfluency measures, namely silent and filled pauses), see also [55] ((number of syllables × 60 s)/total duration), while the articulation rate was calculated by dividing the total number of words by the duration of the phonation time (excluding all disfluency measures) ((number of syllables × 60 s)/duration of phonation).

### 2.4. Statistical Analysis

To identify the possible utility of the four speech-derived indices as classifiers, we followed two statistical approaches: analysis of variance (ANOVA) and hierarchical clustering analysis (HCA). All analyses were performed with OriginPro, version 2022b (OriginLab Corporation, Northampton, MA, USA). Initially, one-way ANOVAs with Tukey’s post hoc tests were performed in order to assess the differences between the four subgroups (healthy and three PPA variants) with regard to the four variables of interest: pause duration and frequency, and speech and articulation rate. The level of statistical significance was set at α = 0.05. Before conducting the ANOVAs, the normality assumption was checked with the Shapiro–Wilk test. Then, we constructed scatter plots with all the possible combinations of pause- and speech-derived variables, in order to preliminary assess the possible groupings based on data visualization. Finally, a set of hierarchical cluster analysis (HCA) procedures were performed for each narrative task, in order to examine the ways that the participants could be re-clustered based on the scores of the speech and pause characteristics. The sets of input variables selected were the same as for the scatter plots (one pause metric and one speech-derived index for each HCA; all possible combinations used). As the official manual of the software mentioned, HCA is appropriate for small samples and starts with each case as a separate cluster, before combining the clusters sequentially and reducing the number of clusters at each step until the desirable number of clusters is reached. The main clustering method uses the dissimilarities between observations when forming the clusters. This approach was expected to provide an estimate on how participants could be clustered and whether any significant discrimination could be determined between healthy participants and PPA patients, as well as between the PPA variants. For the application of the hierarchical cluster analysis, all scores in the four indexes of each task were assumed as input variables. The clustering of observations (participants) was requested. The group average method was selected as the cluster method. The distance type was set to Euclidean, and the clustroids were determined by the sum of distances.

## 3. Results

### 3.1. One-Way ANOVA

**Figure 1:**
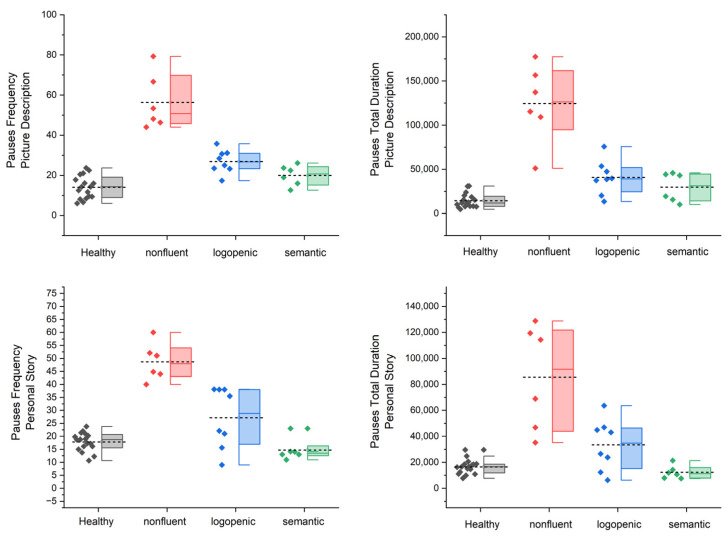
Box plots of pause total duration and frequency data for each type of diagnosis. First row: picture description task. Second row: personal story narration. Each box has a range of 25–75, the whiskers indicate the range of the outliers, the colored line inside each box is the median, and the short dashed lines are the mean.

**Figure 2:**
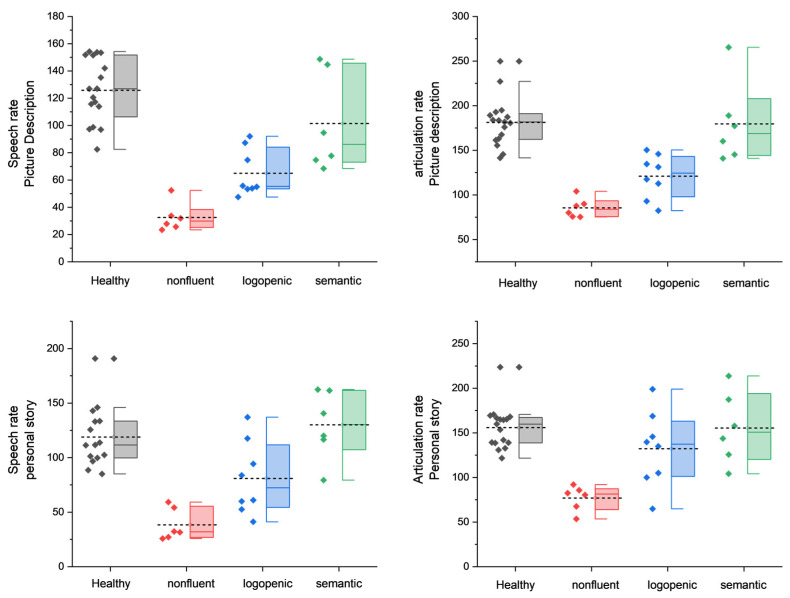
Box plots of speech rate and articulation data for each type of diagnosis. First row: picture description task. Second row: personal story narration. Each box has a range of 25–75, the whiskers indicate the range of the outliers, the colored line inside each box is the median, and the short dashed lines are the mean.

Figure 1 and Figure 2 present the box-plot graphs of each narrative task (picture description and personal story narration) for each of the four speech and pause variables (pause frequency, pause total duration, speech rate, and articulation rate).

**Figure 3:**
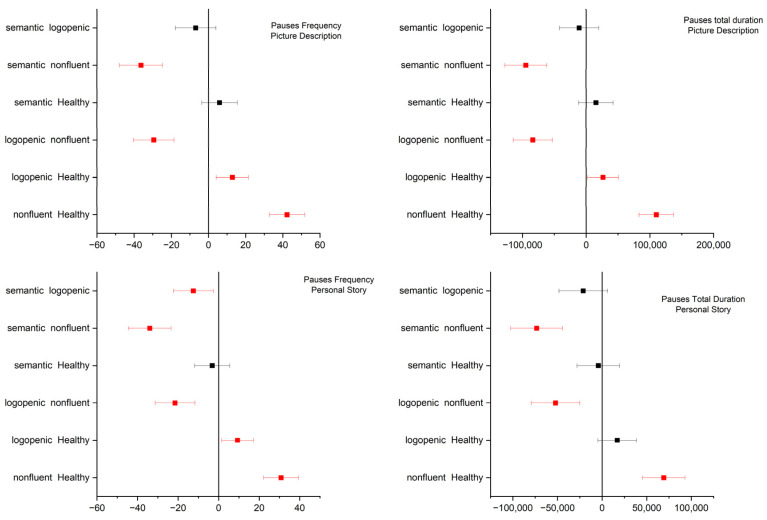
Tukey’s significance test of the one-way ANOVA results for the pause total duration and frequency data of each type of diagnosis. First row: picture description task. Second row: personal story narration. Significance level: 0.05.

**Figure 4:**
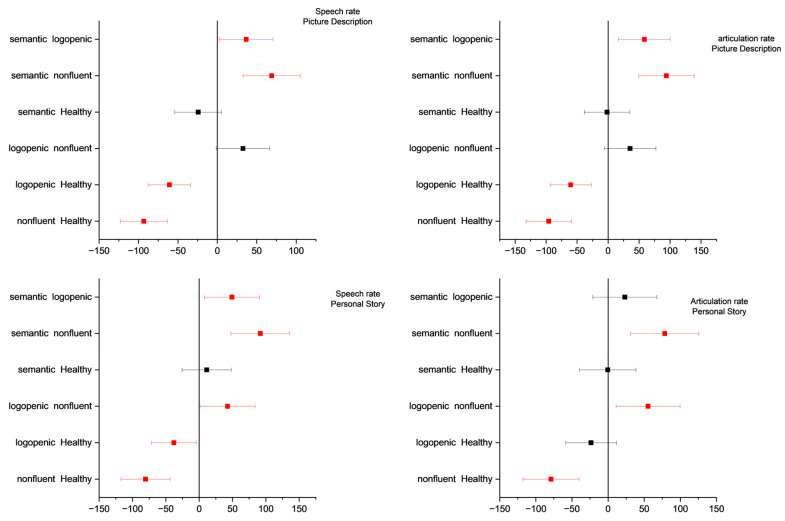
Tukey’s significance test of the one-way ANOVA results for the speech and articulation rate data of each type of diagnosis. First row: picture description task. Second row: personal story narration. Significance level: 0.05.

One-way ANOVA revealed significant differences for all four variables of interest in both elicitation tasks (for the full output of the ANOVA, please see the Supplementary Materials). For picture description, the post hoc pairwise comparisons using Tukey’s significance test revealed significant differences with regard to pause frequency and duration between the semantic and non-fluent, logopenic and non-fluent, logopenic and control, and non-fluent and control groups. Similar results were observed for pauses in the personal story test, with two exceptions: pause frequency was significantly different between the semantic and logopenic groups, while pause total duration was not significantly different between the control and logopenic groups (see Figure 3). Speech and articulation rates in the picture description test exhibited significant differences between the semantic and logopenic, semantic and non-fluent, logopenic and control, and non-fluent and control groups. Similar results were observed for speech rate during the personal story narration, with one additional pair exhibiting significant differences, namely the logopenic and non-fluent groups. With regard to the articulation rate during the personal story narration, there were significant differences between the semantic and non-fluent, logopenic and non-fluent, and non-fluent and control groups (see Figure 4).

**Figure 5:**
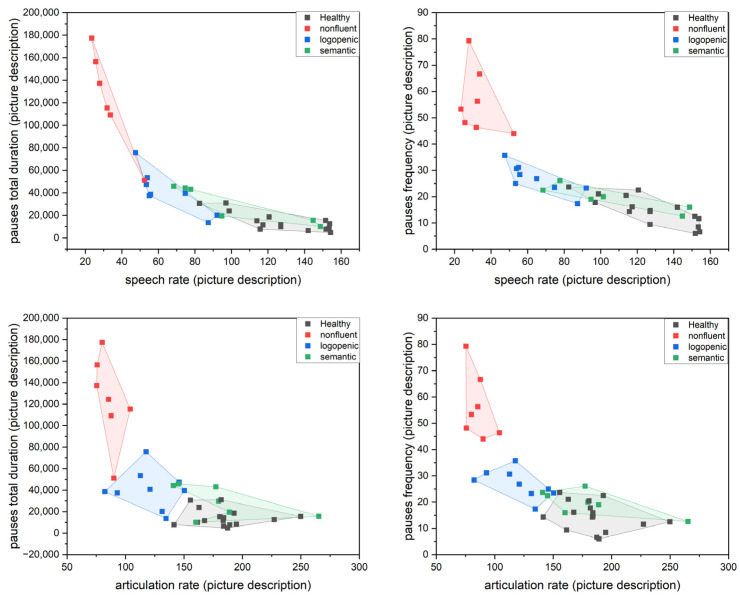
Scatter plots with convex hull ellipse depicting pause attributes versus speech attributes for the picture description task.

**Figure 6:**
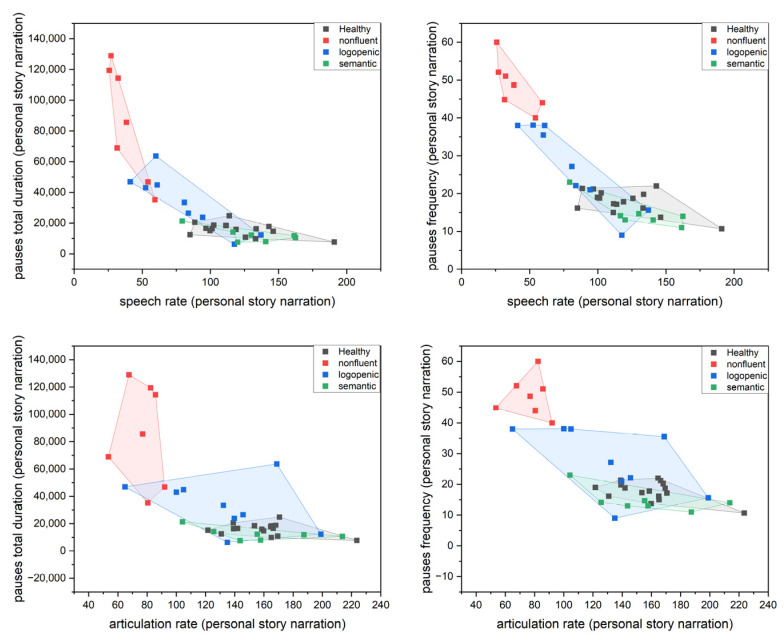
Scatter plots with convex hull ellipse depicting pause attributes versus speech attributes for the personal story task.

The following set of scatter plots was produced. Figure 5 and Figure 6 present the results of pause attributes (duration and frequency) versus speech and articulation rates.

### 3.2. Hierarchical Cluster Analysis

**Figure 7:**
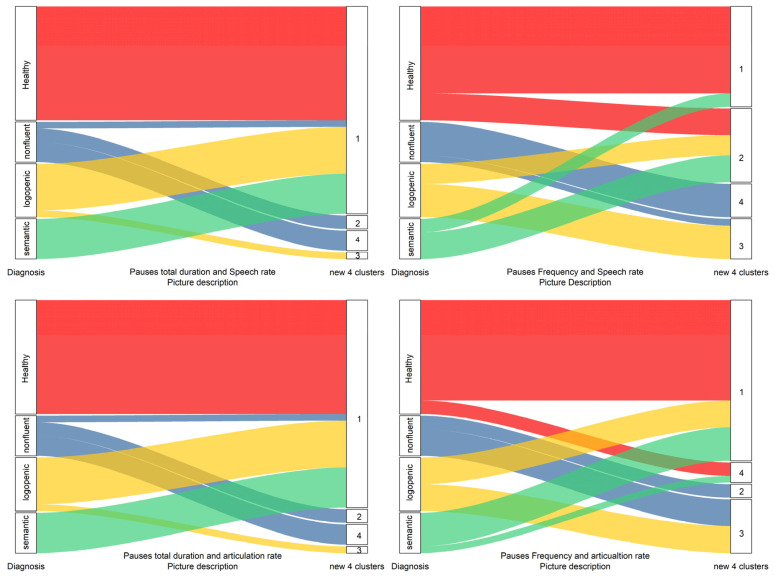
Re-classification of data for the picture description task. First node is the initial diagnosis.

**Figure 8:**
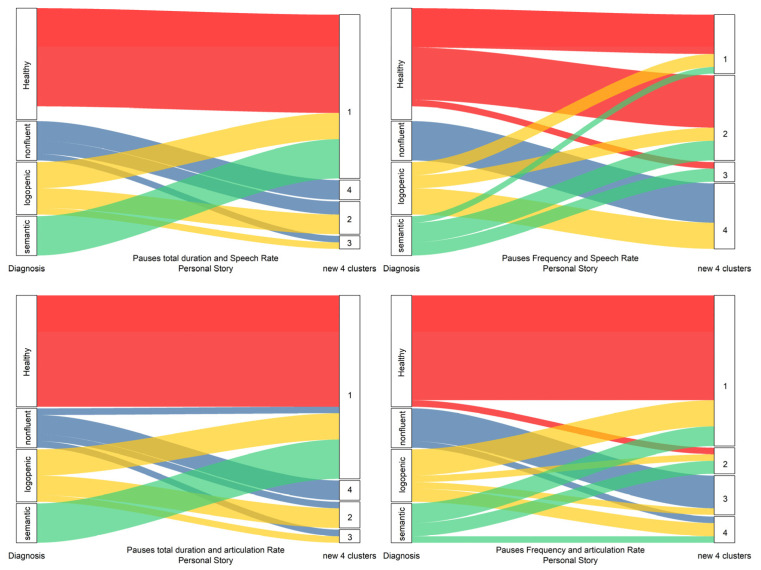
Re-classification of data for the personal story narration task. First node is the initial diagnosis.

As shown in Figure 7 and Figure 8, the hierarchical clustering method performed better for the picture description task than the personal story narration. In the picture narration task cluster analysis, non-fluent patients formed a significantly different cluster of observations (zero similarity) than that formed by other diagnoses or the healthy participants. Moreover, the healthy participants formed another discrete group, different to that of the logopenic or semantic groups. The logopenic and semantic groups were shown earlier to lie between the healthy and non-fluent participants, forming different groups. In contrast, for the personal story narration, the groups formed by the hierarchical cluster analysis were mixed enough to indicate a lower discrimination ability.

## 4. Discussion

One-way ANOVA revealed that pauses and speech indices may have varied between the three variants of PPA and healthy speakers in both tasks. More specifically, non-fluent patients presented a significantly reduced articulation rate compared to healthy speakers as well as the other two PPA variants in both elicitation tasks. Interestingly, the speech rate also appeared to be significantly reduced for non-fluent patients compared to healthy speakers and patients categorized as having the semantic variant in both tasks, but only in the picture description task compared to logopenic patients. With regard to the silent pause indices, non-fluent patients exhibited significantly increased pause frequency and duration compared healthy speakers and the two other PPA variants in both speech genres. Patients falling into the semantic category did not present any significant differences in any index compared to either healthy speakers or patients with the logopenic variant for either elicitation task, while logopenic patients presented significantly reduced articulation and speech rates and increased pause frequency compared to healthy speakers in the picture description task alone.

HCA validated our prior findings and indicated that non-fluent participants could be distinguished from healthy participants and the other PPA variants on the basis of the speech and pause variables derived from the picture description task. On the other hand, the HCA of the personal story narration results revealed lower efficiency with regard to the discrimination between groups. Overall, the HCA results suggested that speech-derived indices and silent pauses—especially those extracted from picture description tasks—may be useful as classifiers; however, the re-classification was far from perfect. We therefore argue that such metrics could be used as complementary biomarkers to efficiently discriminate between PPA patients and healthy speakers, as well as between the three variants of the disease. The observed discrepancy between the two elicitation tasks could be attributed to the different demands on cognitive load, as well as the different hypothesized cognitive mechanisms and neurobiological substrates underlying the performance of each task (for a relevant discussion, see [55]).

Silent pauses have not been widely studied in PPA. Mack and colleagues [41] revealed that silent pause patterns prior to verbs and nouns could be used to differentiate between PPA variants in a narrative task. Therefore, such distinct patterns may reflect different types of underlying impaired mechanisms resulting in word-finding deficits for each PPA variant.

In a more recent study, Nevler and colleagues [30] analyzed pause metrics (frequency and duration) derived from the description of the same picture we used in the current study (the cookie theft picture) in a cohort of PPA patients. Their results revealed that pause frequency could be considered as an effective biomarker for PPA patients, as all three PPA groups differed from healthy speakers with regard to pause frequency. Additionally, non-fluent patients demonstrated a significantly different pattern of pause frequency compared to the other two PPA groups, and logopenic patients differed from the semantic group. However, in contrast to our findings, no significant differences in the mean pause duration appeared between healthy speakers and the PPA groups. It should be noted that we decided to use the total duration of pauses, adjusted for the total number of words uttered, instead of the mean duration as a more representative index for the duration of silent intervals in the speech flow. It could be argued that the two pause metrics, i.e., frequency and duration, may provide different kinds of information. Pause frequency refers to the number of times that a speaker halts and could reflect the processing of new stimuli, an attempt to alter the previously spoken utterance, or the cognitive effort needed to accomplish the originally planned utterance [32]. It could be argued that the sparseness of evidence for a positive correlation between silent pause frequency and naming tasks in PPA patients [41] and patients with Alzheimer’s disease [39] may support this last hypothesis. Silent pause duration, on the other hand, may reflect the efficacy of the aforementioned processing mechanisms, i.e., the amount of time that each individual needs in order to accomplish a certain processing stage while speaking. It should be emphasized that in most studies with a focus on silent pauses, it is usually frequency—and not duration—that is measured. This could be due to the fact that silent pause frequency metrics can be much more easily calculated compared to those related to duration. More studies in both healthy speakers and patients with language disorders are necessary to further clarify the possibly distinct roles of silent pause frequency and duration and their relation to inner cognitive processes while speaking.

In accordance with the above speculations (with regard to the different nature and origin of pause frequency and duration), our results showed that the two pause metrics seemed to have different values as classifiers, as indicated by ANOVA and HCA. Moreover, relevant differentiations were observed between the personal story and picture description tasks. Both findings could be attributed to the aforementioned differences between the two pause variables, but also to the differences between the two elicitation techniques, as mentioned above. Beyond these discrepancies, our results revealed that, independently of the metric or elicitation task, the semantic group could not be differentiated from the healthy participants. This was compatible with García and colleagues’ findings [57], according to which the silent pause mean duration and duration variability (i.e., standard deviation of duration) significantly increased in non-fluent patients compared to healthy speakers and semantic patients, while no differences were detected between the latter two groups. However, it should be noted that this study measured silent pauses using a reading task and not in connected speech.

With regard to speech indices, speech rate has traditionally been considered among the more reliable variables, and it is widely used for the assessment and quantification of speech output in different neurological conditions that result in acquired language deficits, such as primary progressive aphasia [26,27,28] and post-stroke aphasia [55], but also in other neurodegenerative diseases, such as Alzheimer’s disease [58]. Most researchers use speech rate and articulation rate interchangeably to evaluate expressive language abilities. However, only a few studies have indicated the distinct roles of speech and articulation rate in the taxonomy of patients based on their speech output deficits. Cordella and colleagues [59] investigated the diagnostic value of the two speech indices for the three variants of PPA, using speech samples from a picnic scene picture description. Their results revealed that between the two metrics, only the articulation rate was sensitive enough to differentiate the three PPA variants, with non-fluent patients presenting significantly lower index scores compared to the other two variants and healthy speakers, while the speech rate was not significantly different between the non-fluent and logopenic variants. In a later study, Cordella and colleagues [60] elaborated their initial findings by indicating that the articulation rate was significantly reduced compared to the other PPA variants, even during the early stages of the disease, and a longitudinal assessment showed that non-fluent patients presented a greater rate of deterioration compared to semantic and logopenic patients. With regard to the discrepancy between the two speech metrics, similar evidence has arisen for post-stroke aphasia. DeDe and Salis [37] investigated fluency patterns, using both speech rate and articulation rate (defined as pure word rate) in a fairy tale narration exercise, in two cohorts of patients with different post-stroke aphasia profiles: latent aphasia, meaning patients with expressive language difficulties that are not easily detected, and anomic aphasia. Similarly to Cordella and colleagues, their results revealed that the speech rate was significantly reduced in both patient cohorts compared to healthy speakers, yet no significant differences were detected between the two aphasia groups. The articulation rate index, on the other hand, significantly differentiated patients with latent aphasia from patients with anomic deficits. It could be argued that the articulation rate index reflects the motor aspects of speech, such as the articulation complexity of a word and the combination of orofacial muscle movements required to produce specific sounds [61,62], while the involvement of cognitive domains in articulation is still unknown [63]. The speech rate, on the other hand, could be treated as a “mixed” index, as it incorporates both articulation and silent pause duration; thus, it could be argued that it reflects both the motor aspects of speech output and the more cognitive aspects of planning and organizing language expression, as Goldman-Eisler first stated [64,65].

One finding that should be further elaborated is the difference observed between the two elicitation tasks. More specifically, HCA revealed that the speech indices and pause metrics derived from the picture description task were better able to discriminate between the patient groups compared to those derived from the personal story narration. The hypothesis that distinct elicitation techniques represent different cognitive tasks has already been introduced with regard to the investigation of language expression abilities in both healthy speakers and patients with post-stroke aphasia, as well as in patients with mild cognitive impairment and Alzheimer’s disease [36,44,45,46,47,55]. Thus, the performance in terms of the quantification of speech output metrics may be differentiated. In the current study, we selected two speech genres commonly used in speech and language research involving both healthy speakers and patients with acquired language deficits. The picture description task is characterized as a discourse genre of low difficulty with regard to structure, yet due to the fact that the semantic content is predetermined by the visual stimuli, it could be considered of increasing difficulty for patients with word-finding deficits or semantic breakdown. Thus, possible difficulties accessing lexical/semantic representations and phonological representations of words could be better reflected by such tasks [55]. On the other hand, a personal story requires the selective retrieval processing of certain events from the episodic and autobiographic memory, along with their temporal organization, similar to sequencing prior to utterance [55]. Thus, it could be argued that word-finding deficits, which mostly characterize PPA variants, could be better portrayed by indices measured via picture description rather than personal story narration tasks.

The limitations of the current study are related to the relatively small sample size, along with the lack of neuroimaging data and/or CSF biomarkers. Future studies may investigate speech output indices using other elicitation techniques, such as procedural narrative tasks or the narration of stories based on visual stimuli (i.e., the Cinderella story), while possible relationships between the aforementioned speech output indices and atrophy patterns in specific regions of interest could be further assessed.

## 5. Conclusions

PPA is a rather complex clinical entity comprising two different components with regard to the diagnostic pipeline that leads to specific taxonomies: the underlying pathology and the behavioral deficits (the latter being heavily dependent on the type and degree of language impairment). In this context, indices derived from connected speech are of great value, since they have already been shown to be associated with language-related underlying cognitive mechanisms and, most importantly, are of high ecological validity in contrast to the standardized linguistic/psychometric tools and are therefore easily fit into the category of biomarkers, comprising a natural aspect of human behavior. In this paper, we suggest the incorporation of such metrics, namely pause variables and speech/articulation rates, into the diagnostic strategy of PPA, not only as classifiers, but also as quantified indices that would help build a better understanding of the syndrome’s phenotype. We also argue in favor of picture description as being a more adequate elicitation task for this purpose. Finally, we further stress the value of mathematical algorithms in clinical research attempting to create taxonomic categories for complex disorders with highly variable manifestations, such as PPA.

## Figures and Tables

**Table 1:** Demographic characteristics of the participants.

	PPA Patients		Healthy Speakers	
	Range	Mean (SD)	Range	Mean (SD)
Age (years)	52–84	66.13 (8.8)	53–65	58.76 (4.1)
Education (years)	2–18	13.40 (4.5)	6–20	14.58 (4.0)

**Table 2:** Demographic and clinical characteristics of the PPA patients.

Patient No.	Age	Sex	Education (Years)	Hand Preference	TPO in Months	PPA Variant	BNT	BDAE—Oral Expression	BDAE—Auditory Comprehension	Speech Rate (CTP)
P001	84	M	16	Right	21	non-fluent	-	23	29.5	33.70
P002	74	M	12	Right	7	non-fluent	4	20	23	27.84
P008	65	M	14	Right	15	non-fluent	ΝA	19	24	25.70
P010	74	M	2	Right	8	non-fluent	5	-	-	23.50
P018	74	F	18	Right	16	non-fluent	-	23	30.5	52.40
P021	67	M	18	Right	24	non-fluent	10	18	25	31.90
P005	57	M	16	Right	44	semantic	1	28	21.5	68.40
P006	76	M	16	Right	36	semantic	1	18	20.5	74.70
P009	70	M	17	Right	36	semantic	0	26	23.5	144.70
P011	57	M	14	Right	36	semantic	0	21	15	77.70
P012	66	F	16	Right	36	semantic	1	23	24.5	94.70
P014	74	F	17	Ambidextrous	24	semantic	1	23	26	148.70
P007	67	M	12	Right	24	logopenic	11	23	26	92.10
P003	64	M	6	Right	20	logopenic	0	22	22	53.40
P004	76	M	12	Right	12	logopenic	4	23	31	55.71
P013	59	F	16	Right	46	logopenic	6	21	28	87.30
P016	54	F	6	Right	4	logopenic	1	22	31	74.70
P017	61	F	16	Right	10	logopenic	2	23	31	53.90
P020	66	M	17	Right	ΝA	logopenic	14	28	29	47.50
P022	58	M	14	Right	18	logopenic	15	18	27	55.00

## Data Availability

The data presented in this study are available on request from the corresponding author.

## Supplementary Materials

The following supporting information can be downloaded at: https://www.mdpi.com/article/10.3390/medicina58101352/s1, File: ANOVAOneWay.
