# Supplementary material for: Silent Pauses and Speech Indices as Biomarkers for Primary Progressive Aphasia
Source: Medicina (Kaunas). 2022 Sep 27;58(10):1352. doi: 10.3390/medicina58101352 (PMC9611099; doi:10.3390/medicina58101352)
Supplement: Supplementary file 1 [file medicina-58-01352-s001.zip › medicina-1862461-supplementary.pdf]

ANOVAOneWay—Supplementary Material

Input Data

	Data	Range
Dependent Variable	pauses frequency – picture description	[1*:37*]
Factor	Diagnosis	[1*:37*]

Descriptive Statistics

	N Analysis	N Missing	Mean	Standard Deviation	SE of Mean
Healthy	17	0	14,02461	5,6611	1,37302
nonfluent	6	0	56,29999	13,87559	5,66469
logopenic	8	0	26,86889	5,75405	2,03437
semantic	6	0	19,97213	5,06341	2,06713

Overall ANOVA

	DF	Sum of Squares	Mean Square	F Value	Prob>F
Model	3	8100,26596	2700,08865	48,54731	3,3656E-12
Error	33	1835,38326	55,61767		
Total	36	9935,64922			

Null Hypothesis: The means of all levels are equal.
Alternative Hypothesis: The means of one or more levels are different.
At the 0.05 level, the population means are significantly different.

Tukey Test

	MeanDiff	SEM	q Value	Prob	Alpha	Sig	LCL	UCL
nonfluent Healthy	42,27538	3,54136	16,88233	0	0,05	1	32,69618	51,85458
logopenic Healthy	12,84428	3,19747	5,68091	0,00174	0,05	1	4,19528	21,49328
logopenic nonfluent	-29,4311	4,02763	10,33408	1,06792E-7	0,05	1	-40,32563	-18,53656
semantic Healthy	5,94751	3,54136	2,37509	0,35034	0,05	0	-3,63168	15,52671
semantic nonfluent	-36,32787	4,30572	11,93189	0	0,05	1	-47,97461	-24,68112
semantic logopenic	-6,89677	4,02763	2,42165	0,33362	0,05	0	-17,7913	3,99777

Input Data

	Data	Range
Dependent Variable	pauses total duration – Picture Description	[1*:37*]
Factor	Diagnosis	[1*:37*]

Descriptive Statistics

	N Analysis	N Missing	Mean	Standard Deviation	SE of Mean
Healthy	17	0	14434,8259	8067,22619	1956,58975
nonfluent	6	0	124505,85942	44032,71288	17976,27976
logopenic	8	0	40787,76304	19299,42108	6823,37576
semantic	6	0	29794,09106	16353,05349	6676,10613

Overall ANOVA

	DF	Sum of Squares	Mean Square	F Value	Prob>F
Model	3	5,45799E10	1,81933E10	40,89758	3,20259E-11
Error	33	1,46801E10	4,44851E8		
Total	36	6,926E10			

Null Hypothesis: The means of all levels are equal.
Alternative Hypothesis: The means of one or more levels are different.
At the 0.05 level, the population means are significantly different.

Tukey Test

	MeanDiff	SEM	q Value	Prob	Alpha	Sig	LCL	UCL
nonfluent Healthy	110071,03352	10015,45941	15,54237	8,06456E-9	0,05	1	82979,7423	137162,32473
logopenic Healthy	26352,93714	9042,89719	4,12132	0,03076	0,05	1	1892,3756	50813,49867
logopenic nonfluent	-83718,09638	11390,70074	10,39403	9,20834E-8	0,05	1	-114529,34311	-52906,84965
semantic Healthy	15359,26516	10015,45941	2,16878	0,42966	0,05	0	-11732,02606	42450,55637
semantic nonfluent	-94711,76836	12177,17131	10,99949	1,0752E-8	0,05	1	-127650,37663	-61773,1601
semantic logopenic	-10993,67198	11390,70074	1,36492	0,76993	0,05	0	-41804,91871	19817,57475

Input Data

	Data	Range
Dependent Variable	speech rate – Picture Description	[1*:37*]
Factor	Diagnosis	[1*:37*]

Descriptive Statistics

	N Analysis	N Missing	Mean	Standard Deviation	SE of Mean
Healthy	17	0	125,82959	23,23354	5,63496
nonfluent	6	0	32,50667	10,4572	4,26913
logopenic	8	0	64,95125	17,22164	6,08877
semantic	6	0	101,48333	36,11262	14,74292

Overall ANOVA

	DF	Sum of Squares	Mean Square	F Value	Prob>F
Model	3	46991,94245	15663,98082	29,07226	2,1972E-9
Error	33	17780,22584	538,79472		
Total	36	64772,16829			

Null Hypothesis: The means of all levels are equal.
Alternative Hypothesis: The means of one or more levels are different.
At the 0.05 level, the population means are significantly different.

Tukey Test

	MeanDiff	SEM	q Value	Prob	Alpha	Sig	LCL	UCL
nonfluent Healthy	-93,32292	11,02238	11,97369	0	0,05	1	-123,13789	-63,50796
logopenic Healthy	-60,87834	9,95204	8,65099	3,94443E-6	0,05	1	-87,79809	-33,95859
logopenic nonfluent	32,44458	12,53589	3,66018	0,06486	0,05	0	-1,46433	66,3535
semantic Healthy	-24,34626	11,02238	3,12372	0,14189	0,05	0	-54,16122	5,46871
semantic nonfluent	68,97667	13,40143	7,27891	6,80716E-5	0,05	1	32,72651	105,22682
semantic logopenic	36,53208	12,53589	4,1213	0,03076	0,05	1	2,62317	70,441

Input Data

	Data	Range
Dependent Variable	articulation rate – Picture Description	[1*:37*]
Factor	Diagnosis	[1*:37*]

Descriptive Statistics

	N Analysis	N Missing	Mean	Standard Deviation	SE of Mean
Healthy	17	0	181,25705	27,04716	6,5599
nonfluent	6	0	85,45622	10,91896	4,45765
logopenic	8	0	120,96077	24,29866	8,59087
semantic	6	0	179,62652	45,82654	18,70861

Overall ANOVA

	DF	Sum of Squares	Mean Square	F Value	Prob>F
Model	3	53400,30263	17800,10088	21,8088	5,7209E-8
Error	33	26934,23158	816,18884		
Total	36	80334,53421			

Null Hypothesis: The means of all levels are equal.
Alternative Hypothesis: The means of one or more levels are different.
At the 0.05 level, the population means are significantly different.

Tukey Test

	MeanDiff	SEM	q Value	Prob	Alpha	Sig	LCL	UCL
nonfluent Healthy	-95,80083	13,56623	9,98677	2,37793E-7	0,05	1	-132,49677	-59,10489
logopenic Healthy	-60,29628	12,24887	6,96161	1,31051E-4	0,05	1	-93,42882	-27,16374
logopenic nonfluent	35,50455	15,42903	3,25432	0,11837	0,05	0	-6,23017	77,23928
semantic Healthy	-1,63053	13,56623	0,16997	0,99937	0,05	0	-38,32647	35,06541
semantic nonfluent	94,1703	16,49433	8,0741	1,30728E-5	0,05	1	49,554	138,7866
semantic logopenic	58,66575	15,42903	5,37726	0,00314	0,05	1	16,93102	100,40047

Input Data

	Data	Range
Dependent Variable	pauses frequency – Personal story	[1*:37*]
Factor	Diagnosis	[1*:37*]

Descriptive Statistics

	N Analysis	N Missing	Mean	Standard Deviation	SE of Mean
Healthy	17	0	17,84361	3,55792	0,86292
nonfluent	6	0	48,65876	7,17476	2,92908
logopenic	8	0	27,16367	11,65097	4,11924
semantic	6	0	14,69024	4,22283	1,72396

Overall ANOVA

	DF	Sum of Squares	Mean Square	F Value	Prob>F
Model	3	4888,35861	1629,45287	35,86462	1,7005E-10
Error	33	1499,30349	45,43344		
Total	36	6387,6621			

Null Hypothesis: The means of all levels are equal.
Alternative Hypothesis: The means of one or more levels are different.
At the 0.05 level, the population means are significantly different.

Tukey Test

Tukey Test

	MeanDiff	SEM	q Value	Prob	Alpha	Sig	LCL	UCL
nonfluent Healthy	30,81516	3,20075	13,61532	1,00099E-7	0,05	1	22,1573	39,47301
logopenic Healthy	9,32006	2,88994	4,56085	0,01435	0,05	1	1,50294	17,13719
logopenic nonfluent	-21,49509	3,64025	8,3507	7,3594E-6	0,05	1	-31,34178	-11,64841
semantic Healthy	-3,15337	3,20075	1,39328	0,75878	0,05	0	-11,81123	5,50449
semantic nonfluent	-33,96853	3,89159	12,34425	8,22798E-9	0,05	1	-44,49508	-23,44198
semantic logopenic	-12,47344	3,64025	4,84585	0,00856	0,05	1	-22,32012	-2,62675

Input Data

	Data	Range
Dependent Variable	pauses total duration – personal story	[1*:37*]
Factor	Diagnosis	[1*:37*]

Descriptive Statistics

	N Analysis	N Missing	Mean	Standard Deviation	SE of Mean
Healthy	17	0	16439,90813	5438,10544	1318,9343
nonfluent	6	0	85605,37045	40410,35248	16497,45732
logopenic	8	0	33413,48941	19437,12084	6872,05998
semantic	6	0	12261,4798	5093,62379	2079,4632

Overall ANOVA

	DF	Sum of Squares	Mean Square	F Value	Prob>F
Model	3	2,36432E10	7,88108E9	22,78868	3,54384E-8
Error	33	1,14125E10	3,45833E8		
Total	36	3,50557E10			

Null Hypothesis: The means of all levels are equal.
Alternative Hypothesis: The means of one or more levels are different.
At the 0.05 level, the population means are significantly different.

Tukey Test

	MeanDiff	SEM	q Value	Prob	Alpha	Sig	LCL	UCL
nonfluent Healthy	69165,46232	8830,73839	11,07662	5,73881E-9	0,05	1	45278,77919	93052,14546
logopenic Healthy	16973,58128	7973,21981	3,01061	0,16513	0,05	0	-4593,55917	38540,72173
logopenic nonfluent	-52191,88104	10043,30348	7,34922	5,88526E-5	0,05	1	-79358,48901	-25025,27308
semantic Healthy	-4178,42833	8830,73839	0,66916	0,96446	0,05	0	-28065,11147	19708,25481
semantic nonfluent	-73343,89065	10736,74305	9,66065	4,80938E-7	0,05	1	-102386,21619	-44301,56512
semantic logopenic	-21152,00961	10043,30348	2,97845	0,17225	0,05	0	-48318,61758	6014,59836

Input Data

	Data	Range
Dependent Variable	speech rate – personal story	[1*:37*]
Factor	Diagnosis	[1*:37*]

There exist missing values in the input data.

Descriptive Statistics

	N Analysis	N Missing	Mean	Standard Deviation	SE of Mean
Healthy	15	2	118,87011	27,51971	7,10556
nonfluent	6	0	38,38333	14,53512	5,93394
logopenic	8	0	80,9375	33,61284	11,88393
semantic	6	0	130,06667	31,63667	12,91562

Overall ANOVA

	DF	Sum of Squares	Mean Square	F Value	Prob>F
Model	3	36353,344	12117,78133	15,28766	2,7664E-6
Error	31	24572,18299	792,65106		
Total	34	60925,52699			

Null Hypothesis: The means of all levels are equal.
Alternative Hypothesis: The means of one or more levels are different.

At the 0.05 level, the population means are significantly different.

Tukey Test

	MeanDiff	SEM	q Value	Prob	Alpha	Sig	LCL	UCL
nonfluent Healthy	-80,48678	13,5997	8,3697	8,88001E-6	0,05	1	-117,39735	-43,5762
logopenic Healthy	-37,93261	12,32578	4,35225	0,02141	0,05	1	-71,38565	-4,47956
logopenic nonfluent	42,55417	15,20493	3,95797	0,04129	0,05	1	1,28689	83,82144
semantic Healthy	11,19656	13,5997	1,16431	0,843	0,05	0	-25,71401	48,10713
semantic nonfluent	91,68333	16,25475	7,97673	1,95874E-5	0,05	1	47,56676	135,7999
semantic logopenic	49,12917	15,20493	4,56951	0,01469	0,05	1	7,86189	90,39644

Input Data

	Data	Range
Dependent Variable	articulation rate – personal story	[1*:37*]
Factor	Diagnosis	[1*:37*]

Descriptive Statistics

	N Analysis	N Missing	Mean	Standard Deviation	SE of Mean
Healthy	17	0	155,89221	23,59668	5,72303
nonfluent	6	0	76,95149	14,02638	5,72625
logopenic	8	0	132,25786	42,01267	14,85372
semantic	6	0	155,39384	40,1916	16,40815

Overall ANOVA

	DF	Sum of Squares	Mean Square	F Value	Prob>F
Model	3	29882,43361	9960,8112	10,83953	4,13354E-5
Error	33	30324,82108	918,93397		
Total	36	60207,25469			

Null Hypothesis: The means of all levels are equal.
Alternative Hypothesis: The means of one or more levels are different.

At the 0.05 level, the population means are significantly different.

Tukey Test

	MeanDiff	SEM	q Value	Prob	Alpha	Sig	LCL	UCL
nonfluent Healthy	-78,94072	14,39481	7,7555	2,53405E-5	0,05	1	-117,87793	-40,00351
logopenic Healthy	-23,63435	12,99699	2,57167	0,283	0,05	0	-58,79052	11,52182
logopenic nonfluent	55,30637	16,37139	4,77754	0,0097	0,05	1	11,02262	99,59011
semantic Healthy	-0,49837	14,39481	0,04896	0,99998	0,05	0	-39,43558	38,43884
semantic nonfluent	78,44235	17,50175	6,33846	4,68173E-4	0,05	1	31,10103	125,78367
semantic logopenic	23,13598	16,37139	1,99856	0,50032	0,05	0	-21,14777	67,41973
